# Serum CXCL8 and Its Specific Receptor (CXCR2) in Gastric Cancer

**DOI:** 10.3390/cancers13205186

**Published:** 2021-10-15

**Authors:** Elżbieta Pawluczuk, Marta Łukaszewicz-Zając, Mariusz Gryko, Agnieszka Kulczyńska-Przybik, Barbara Mroczko

**Affiliations:** 1 Department of Neurodegeneration Diagnostics, Medical University of Bialystok, 15-269 Bialystok, Poland; elzbieta.pawluczuk16@wp.pl (E.P.); agnieszka.kulczynska-przybik@umb.edu.pl (A.K.-P.); 2 Department of Biochemical Diagnostics, Medical University of Bialystok, 15-269 Bialystok, Poland; marta.lukaszewicz-zajac@umb.edu.pl; 3 Second Department of General Surgery, Medical University of Bialystok, 15-274 Bialystok, Poland; mariusz_gryko@vp.pl

**Keywords:** chemokines, gastric cancer, receptors for chemokines

## Abstract

**Simple Summary:**

Gastric cancer (GC) is a serious medical problem; thus, there is a need for the improvement of the diagnostic process of GC patients because this malignancy is still detected at a late stage of the disease. It is suggested that selected chemokines such as CXCL8 and its specific receptor CXCR2 are involved in cancer progression, including GC. Therefore, we assessed the serum levels of these proteins in 98 subjects: 64 patients with GC and 34 healthy volunteers. The aim of our study was to evaluate the usefulness of serum CXCL8 and CXCR2 concentrations as biomarkers in the diagnosis and progression of this cancer. Our findings suggest that serum CXCL8 might be used as a potential biomarker in the diagnosis of GC patients, especially in combined assessments with classical tumor markers. Our results indicate the role of the CXCL8/CXCR2 axis as well as the inflammation in the pathogenesis of this malignancy. Moreover, serum CXCL8 was the significant predictor of GC risk.

**Abstract:**

Gastric cancer (GC) is the second leading cause of cancer-related deaths worldwide. This malignancy is usually diagnosed at an advanced stage. Therefore, novel biomarkers useful in the early detection of GC are sorely needed. Some authors suggest the role of chemokines and their specific receptors in GC pathogenesis. The aim of the study was to investigate whether serum CXCL8 and its receptor (CXCR2) might be considered as potential candidates for biomarkers in the diagnosis and prognosis of GC. The study included 98 subjects: 64 GC patients and 34 healthy volunteers. CXCL8 and CXCR2 concentrations were assessed by the enzyme-linked immunosorbent assay (ELISA) method. Serum CXCL8 and CXCR2 concentrations were significantly higher in GC patients than in healthy controls, similar to the well-established tumor marker (CA19-9) and marker of inflammation (CRP). Diagnostic sensitivity of CXCL8 was the highest among all proteins tested and increased for the combined assessment with CA19-9. The area under the ROC curve for CXCL8 was higher than those for CXCR2 and classical tumor markers. Serum CXCL8 levels were indicated as a significant risk factor of GC occurrence. Our findings suggest that serum CXCL8 is a promising candidate for a biomarker in GC diagnosis and might be used as a significant predictor of GC risk.

## 1. Introduction

Gastric cancer (GC) is the fifth most commonly diagnosed malignancy worldwide and the fourth most common cancer among men and the sixth in women, according to data published by the World Health Organization (WHO) in 2020. Moreover, it is the third leading cause of cancer-related death [1]. The five-year survival rate for GC patients is lower than 30% [2]. The early stage of this disease is usually asymptomatic or the symptoms are not specific; thus, the diagnostic process is unnecessarily prolonged. When the stage of the cancer is more advanced, other symptoms may occur such as abdominal pain, weight loss or hematemesis. Approximately 80% of GC patients are diagnosed in the advanced stage, when the possibilities of the treatment are limited [3]. The main risk factors for this malignancy are: Helicobacter pylori (H. pylori) infection, smoking, alcohol abuse, increased salt intake, obesity, blood group A or GC in family history [4].

Chemokines are small proteins involved in an inflammation process, autoimmune and cardiovascular diseases as well as malignant diseases [5,6]. These proteins may also lead to tumorigenesis, metastasis, angiogenesis, proliferation and protection from the host response [6,7]. Chemokines are grouped into four classes: CC, XC, CXC and CX3C, where C stands for the key cysteine and X for amino acid [3]. Chemokines, especially the CXC family and their receptors, have an important role in GC pathogenesis and may be used as biomarkers of tumor development and progression in the future [8].

CXCL8 is a chemokine from the CXC group that is also known as interleukin-8 (IL-8). Cells that produce CXCL8 have origins in epithelial and endothelial tissue. CXCL8 is also synthesized by monocytes, macrophages and fibroblasts [9,10]. The secretion of this protein is stimulated by hypoxia, reactive oxygen species (ROS) and interleukins: IL-1, IL-6, IL-22 and chemokine CXCL12 [9]. The presence of H. pylori infection and gastritis may lead to CXCL8 synthesis by stomach epithelial cells. In patients with chronic gastritis, CXCL8 may also be secreted by neutrophils in the stomach lamina propria [8,9]. CXCL8 recruits neutrophils but these cells cannot eliminate H. pylori infection, causing chronic neutrophil inflammation and gastritis. Subsequently, chronic inflammation may contribute to the development of GC. Neutrophils and myeloid-derived suppressor cells (MDSCs) might be recruited to the tumor, that can promote tumor cell proliferation, vascular endothelial growth factor (VEGF) expression and neoangiogenesis [10].

It has been proved that CXCL8 can exist in two forms, as a monomer and as a dimer. As a monomer, CXCL8 binds only to the chemokine receptor type 1 (CXCR1), while both monomers and dimers interact with the chemokine receptor type 2 (CXCR2) [9]. Some clinical investigations have proved that CXCR1 and CXCR2 are associated with a poor GC prognosis [11]. Both receptors have a 76% common sequence and similar affinity in attaching to CXCL8 [9]. The difference is that CXCR1 weakly binds to other chemokines, whereas CXCR2 interacts with CXCL1, CXCL2, CXCL3, CXCL5, CXCL6, CXCL7 and CXCL8 [9,12]. CXCR1 and CXCR2 are seven transmembrane G protein-coupled receptors [13]. The overexpression of CXCR1 and CXCR2 was associated with an advanced GC stage and the presence of distant metastasis [14].

The diagnosis of GC is established based on invasive methods. One of them is gastroscopy with a biopsy and histopathological examination. Additional methods are computed tomography (CT), magnetic resonance imaging (MRI) and endoscopic ultrasound scanning (EUS) [15]. Moreover, the measurement of biochemical tumor markers such as cancer antigen 72-4 (CA72-4), carcinoembryonic antigen (CEA) or carbohydrate antigen (CA19-9) concentrations are also very important in the diagnosis of patients with this malignancy; however, these biomarkers cannot be used in the early detection of GC. Thus, there is a need for novel blood biomarkers to improve the diagnostic process, especially the early detection of this disease and escalate the treatment chances as well as the number of cancer survivors [16,17,18]. Therefore, the aim of the study was to investigate whether serum levels of CXCL8 and its specific receptor CXCR2 may be used as potential biochemical markers for GC. Based on our knowledge, the present study is the first to compare the significance of these proteins in relation to the well-established tumor markers (CEA and CA19-9) and the marker of inflammation—C-reactive protein (CRP)— in the diagnosis and progression of GC. Moreover, the present paper is a continuation of our previous research, in which we assessed that serum concentrations of selected chemokines and their specific receptors might be used as potential tumor biomarkers for gastrointestinal malignancies, including pancreatic, esophageal and colorectal cancer [19,20,21,22,23,24,25].

## 2. Materials and Methods

The study group consisted of 98 patients—64 GC patients (41 males and 23 females, aged from 28 to 82 years) and 34 healthy volunteers with a negative history of inflammatory diseases and cancers (24 males and 10 females, aged from 27 to 76) as the control group. GC patients were diagnosed and operated on at the Second Department of General Surgery, Medical University of Bialystok (Poland). The microscopic examination of material obtained during biopsy and/or surgery was used in the clinical diagnosis of GC.

The GC group was divided into 4 subgroups regarding the GC stage: stage I, stage II, stage III and stage IV. The GC patients were staged according to the TNM Classification of Malignant Tumors UICC (TNM—depth of tumor invasion, lymph nodes metastasis, distant metastasis) by the International Union Against Cancer (Genève, Switzerland UICC). Moreover, features such as Lauren classification and grading were also evaluated. The characteristics of GC patients are presented in Table 1.

The project was approved by the Local Ethics Committee (R-I-002/65/2017) of the Medical University of Bialystok (Bialystok, Poland). All patients and healthy volunteers were granted informed consent to participate in the study.

Blood samples were taken from GC patients before the treatment and frozen at −80 °C. Serum levels of CXCL8 (Quantikine ELISA Human CXCL8/IL8 Immunoassay, R&D Systems, Abingdon, UK) and CXCR2 (EIAab, Wuhan, China) were assessed with the enzyme-linked immunosorbent assay (ELISA) according to the manufactures’ instructions. Concentrations of classical cancer markers such as carcinoembryonic antigen (CEA) and carbohydrate antigen 19-9 (CA19-9) were measured by the chemiluminescent microparticle immunoassay (CMIA) on the ARCHITECT 8200 ci (Abbott, Chicago, IL, USA), while serum C-reactive protein (CRP) concentrations using turbidimetric method also on ARCHITECT 8200 ci (Abbott, Chicago, IL, USA).

In order to select the optimal predicted probability cut-off values, Youden’s index was used. The reference cut-off values were as follows: 23.11 pg/mL for CXCL8; 1.15 ng/mL for CXCR2; 4.65 ng/mL for CEA; 5.59 U/mL for CA19-9; and 5.50 mg/L for CRP.

## 3. Statistical Analysis

Distributions of serum concentrations of all analyzed proteins (CXCL8, CXCR2, CA19-9, CEA and CRP) both in GC patients and control group were assessed using the Shapiro–Wilk test, with conclusions that the normal distribution hypothesis has to be rejected; thus, non-parametric statistical analyses were performed.

For two-groups analysis, the Mann–Whitney test was used, while in the case of three or more groups to compare, the Kruskal–Wallis test was exploited. Further analysis of groups with statistically significant differences was conducted with the use of a post hoc Dwass–Steele–Critchlow–Fligner test. Correlations between parameters were calculated utilizing Spearman’s rank correlation test. The calculated differences were found statistically significant if the *p* value was less than 0.05.

The assessment of diagnostic usefulness of the analyzed proteins was made based on diagnostic sensitivity, specificity, accuracy and positive and negative predictive values. The Receiver Operating Characteristic (ROC) curves analysis was also calculated.

Statistical analysis was mostly made in IBM SPSS Statistics 20.0 software, while additional results processing, like ROC curves analysis and calculating diagnostic parameters of tests, were performed in Microsoft Office Excel.

Logistic regression was used to assess correlations between risk factors and GC. Univariate logistic regression models were obtained for each risk factor and multivariate analyses were employed for variables *p* < 0.05.

## 4. Results

Serum levels of CXCL8, CXCR2, CRP, CA19-9 and CEA were measured and compared between the two analyzed groups—the GC patients group and the control group, as shown in Table 2. Concentrations of CXCL8 were significantly higher in GC patients than in the control group (28.486 vs. 6.561 pg/mL, *p* < 0.001). Moreover, serum CXCR2 levels were also significantly elevated in GC patients when compared to healthy volunteers (1.449 vs. 0.638 ng/L; *p* < 0.001). The concentrations of classical biomarkers—CA19-9 (7.875 vs. 4.965 U/mL. *p* = 0.009) and CEA (1.67 vs. 1.46 ng/mL; *p* = 0.797) as well as CRP (16.4 vs. 1.05 mg/L; *p* < 0.001) were higher in the cancer group than in healthy volunteers, but only in the case of CEA was the difference not statistically significant (Table 2).

Table 3 represents the serum concentrations of biomarkers in relation to TNM stage and clinicopathological characteristics of the tumor. The highest concentrations of CXCL8 were observed in stage I of GC (33.089 pg/mL), while CXCR2 (1.823 ng/mL) and CA19-9 (14.815 U/mL) levels were the highest in stage III of the disease. In addition, CRP (52.9 mg/L) and CEA (1.810 ng/mL) concentrations were the highest in stage IV of GC; however, the statistically significant differences between tumor stages were found only for CRP concentrations (*p* = 0.044). Moreover, serum levels of CXCL8, CXCR2 and classical tumor markers were higher in Lauren type 2 than in patients with Lauren type 1, while CRP concentrations were the highest in patients with Lauren type 1. Interestingly, CXCL8 and CXCR2 concentrations were found to be the highest in patients with a greater depth of tumor invasion (T4 stage) in comparison to T1 + 2 and T3 patients and likewise with the levels of classical tumor markers (CEA and CA19-9) and CRP; however, these differences were statistically significant only for CRP concentrations (*p* = 0.020) in the Kruskal–Wallis test. Moreover, serum CA19-9 levels were significantly elevated in T4 subgroup in comparison to T1 + 2 subjects (*p* = 0.031) (Table 3). If we consider the relationship between serum levels of analyzed proteins and the presence of lymph node and distant metastases (N and M factor), CXCL8 concentrations were the highest in patients with nodal involvement (N3 subgroup) when compared to N0 and N1 + 2 patients. Similar results were also observed for CRP, CEA and CA19-9 levels; however, a statistically significant difference was found for CA19-9 levels (*p* = 0.034). Serum CXCR2 levels were the most elevated in N1 + 2 patients, but these differences were not statistically relevant. There were also no significant differences between CXCL8, CXCR2 and classical tumor markers levels and the presence of distant metastasis (M factor). Serum CRP levels were significantly elevated in patients with distant metastases than in subjects from the M0 subgroup (*p* = 0.048) (Table 3).

### 4.1. Correlations between Serum Concentrations and Gastric Cancer Features

Spearman’s rank correlation test was performed to detect relations between serum concentrations of all analyzed proteins and selected GC features (Table 4). The serum CXCL8 concentration was found to be significantly correlated with its specific receptor CXCR2 concentration (r = 0.57, *p* < 0.001) as well as with CRP (r = 0.58, *p* < 0.001) and CA19-9 levels (r = 0.21, *p* = 0.042), whereas CXCR2 levels were associated with CRP concentrations (r = 0.54, *p* < 0.01).

The relationship between chosen risk factors (serum CXCL8 and its specific receptor —CXCR2 as well as classical tumor markers and CRP) and GC risk was examined using univariate analysis to assess the risk factors that subsequently were employed in the multivariate model. Serum concentrations of CXCL8 (*p* < 0.001, OR = 1.125), CXCR2 (*p* < 0.001, OR = 3.923) and CRP (*p* = 0.005, OR = 1.457) were associated with a significantly increased risk of GC occurrence. Thus, these variables were included in the multivariate analysis. Ultimately, serum CXCL8 (*p* = 0.002, OR = 1.137) and CRP (*p* = 0.041, OR = 1.279) concentrations were indicated to be significant risk factors of GC occurrence (Table 5).

### 4.2. Diagnostic Usefulness of CXCL8 and CXCR2 as Gastric Cancer Biomarkers

According to the aim of the study, the assessment of diagnostic usefulness of serum CXCL8 and CXCR2 measurement in GC patients was performed basing on calculated diagnostic sensitivity, specificity, accuracy (ACC), positive predictive value (PPV) and negative predictive value (NPV) as well as the areas under ROC curves (AUC). The results are presented in Table 6.

The diagnostic sensitivity of CXCL8 (67%) was higher than for its receptor, CXCR2 (63%) and CEA (20%), and comparable to CRP (67%) and CA19-9 (70%) levels. The combined measurements of CXCL8 with classic tumor biomarkers increased the diagnostic sensitivity up to 89%, when CXCL8 was analyzed together with CA19-9. The diagnostic specificity of CXCL8 was higher (94%) in comparison to CA19-9 (59%), but lower than for CEA and CRP levels (both 100%). Moreover, positive predictive value (PPV) levels for CXCL8 (96%) and CXCR2 (89%) were higher than those of CA19-9 (76%) and lower when compared to CEA and CRP (both 100%). The combined measurements of CXCL8 and other proteins did not increase PPV value. The assessment of CXCR2 with all other proteins increased PPV values in comparison to individual PPV of CXCR2 (89%). Negative predictive value (NPV) concentrations for CXCL8 (60%) and CXCR2 (55%) were also higher than CA19-9 (51%) and CEA (40%). The highest NPV values were recorded for the combined measurements of CXCR2 with CA19-9 (76%) and CXCL8 with CRP (74%) levels. The diagnostic accuracy (ACC) of CXCL8 (77%) was higher than CXCR2 (70%) and classical tumor markers, CEA (48%) and CA19-9 (66%), but lower than for CRP (79%). The ACC values of combined measurements of CXCL8 with CRP and CXCR2 with CA19-9 were found to be the highest (both 87%) (Table 6).

**Figure 1:**
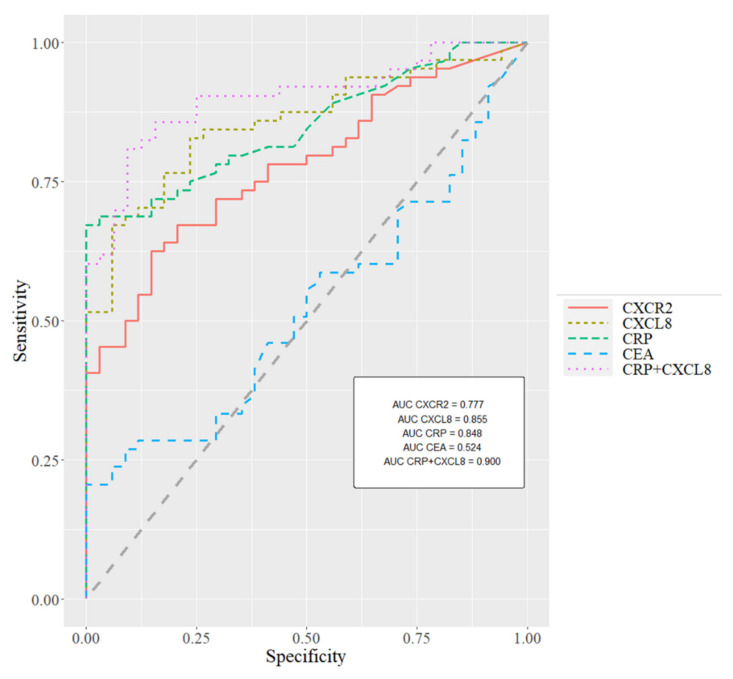
The areas under ROC (Receiver Operating Characteristic) curves for biomarkers tested in gastric cancer.

To evaluate the diagnostic significance of CXCL8 and its receptor as candidates for GC biomarkers, the areas under ROC curves (AUC) were also calculated. ROC curves for all biomarkers are visualized in Figure 1. The AUC for CXCL8 (0.8552, *p* < 0.001) was higher than the AUC for its receptor CXCR2 (AUC = 0.7773, *p* < 0.001) and classical tumor markers (CEA—AUC = 0.5159 and CA19-9—AUC = 0.6606), and also higher than the AUC for CRP levels (AUC = 0.8488, *p* < 0.001). Moreover, the combined analysis of CXCL8 and CRP increased the AUC to 0.900 (*p* < 0.001) (Figure 1).

## 5. Discussion

GC is a serious medical problem. According to WHO statistics, GC accounted for 5.6% of all new cancer cases in 2020. The five-year prevalence of this cancer is 23.70 per 100,000 individuals [1]. Thus, there is a need for the improvement of the diagnostic process and non-invasive methods such as the measurement of new biomarkers concentrations because GC is still diagnosed at a late stage. Many researchers found that selected chemokines such as CXCL8 and its specific receptor CXCR2 are involved in cancer progression via the stimulation of tumor invasion, angiogenesis and metastasis. Some clinical investigations suggest that CXCL8 plays a significant role in cancer development, including GC [10]. A growing body of evidence indicated that the CXCR2 receptor interacts with several chemokines including CXCL1, -2, -3, -5, -6, -7 as well as with CXCL8 [26]. Some clinical investigations suggest that CXCL8 and its receptor CXCR2 might be candidates for biomarkers for GC. Therefore, the aim of this study was to assess the potential significance of CXCL8 and CXCR2 in the diagnosis and prognosis of GC and their role in the pathogenesis of this malignancy.

In our study, we revealed that the concentrations of CXCL8 and CXCR2 were significantly higher in GC patients than in healthy controls, which may suggest that GC cells are involved in the synthesis of these proteins. Similar findings were observed by Baj-Krzyworzeka, who also indicated that CXCL8 levels in GC patients were significantly higher than in the control group [7], which was also confirmed by other authors [27,28]. Our previous investigations indicated the significant differences between serum levels of CXCL8 and/or CXCR2 in cancer patients and healthy individuals; however, these studies were performed on pancreatic [22,23], colorectal [24,25] as well as esophageal cancer [20,21] patients.

In the present investigation, there were no statistically significant differences between TNM stages of GC in the CXCL8 and CXCR2 serum levels. Wang et al. indicated that the elevated expression of CXCR2 was associated with tumor depth, nodal involvement and advanced TNM stage [13], while Li et al. found that higher CXCR2 expression in GC was associated with the presence of lymph node and distant metastases and advanced clinical stage [14]. Opposite results were presented by Chen et al., who demonstrated that a reduced expression of CXCR2 was correlated with larger tumor size and advanced TNM stage [8]. Moreover, Baj-Krzyworzeka et al. noticed that the plasma concentration of CXCL8 in GC patients also increased with the more advanced stage of the disease [7]. Moreover, in our previous studies, there were significant differences between CXCL8 concentrations and nodal involvement in patients with pancreatic cancer [22], while the serum level of this chemokine was statistically higher in subjects with distant metastases than without them in CRC patients [24]. The lack of statistical differences in serum CXCL8 and CXCR2 levels between the TNM stage and clinicopathological characteristics of the tumor might be a result of different types of samples and methods employed in presented investigations as well as an insufficient number of patients in specific subgroups included in our research.

Currently, using Spearman’s rank correlation test results, we indicated that serum CXCL8 concentrations significantly correlated with its specific receptor CXCR2 and CRP and CA19-9 levels, whereas CXCR2 levels were associated with CRP concentrations. Present observations were in line with our previous studies concerning the measurement of these proteins in the sera of patients with gastrointestinal cancer. Preoperative serum CRC concentrations of CXCL8 were correlated with CEA and CRP levels as well as with the presence of distant metastases [24]. In addition, there was a significant correlation between serum CXCL8 concentration and CRP levels as well as with nodal involvement in pancreatic cancer patients [22]. Moreover, the serum CXCL8 level was indicated to be a significant risk factor of GC occurrence. Our previous study also revealed that this chemokine was the only significant predictor of pancreatic cancer risk [22].

In our study, we compare the diagnostic criteria for the measurement of the serum CXCL8 and its receptor levels with classical biochemical markers and markers of inflammation. The diagnostic sensitivity of CXCL8 was higher than for its receptor CXCR2 and CEA and comparable to CRP and CA19-9 levels. The highest diagnostic sensitivity and PPV were assessed for a combined analysis of CXCL8 with CA19-9. In addition, the diagnostic specificity of CXCL8 was higher when compared to CXCR2 and CA19-9 but lower than CEA and CRP. NPV values for CXCL8 and CXCR2 were higher in comparison to those of classical tumor markers. Moreover, AUC for CXCL8 was higher than for CXCR2 and classical tumor markers. Based on the literature database, there is no study comparing the diagnostic criteria for serum CXCL8 and CXCR2 levels with well-established tumor markers and CRP as GC biomarkers. In our previous findings, the percentages of elevated results for CXCL8 and CXCR2 were higher than for the classical tumor marker and the combined analysis of both proteins also increased the diagnostic sensitivity, which may suggest that it would be more useful to measure these proteins together than with a single marker in the diagnosis of colorectal cancer patients [24,25]. Similar to our present observations, the highest sensitivity was seen in the combined analysis of a protein tested with a classical tumor biomarker [20,21,22,23,24,25]. Our investigations revealed that the assessment of a single biomarker is not accurate enough to be used as a diagnostics tool because of its non-specific nature.

## 6. Conclusions

The findings presented in this paper suggest that based on diagnostic characteristics of all proteins tested, serum CXCL8 might be used as a potential biomarker in the diagnosis of GC patients, especially in a combined assessment with classical tumor markers. Our results indicate the role of the CXCL8/CXCR2 axis as well as the inflammation in the pathogenesis of this malignancy. Moreover, serum CXCL8 was a significant predictor of GC risk.

## Figures and Tables

**Table 1:** Characteristics of gastric cancer patients.

Gastric Cancer Patients		64
Gender	Female	23
	Male	41
Age	Median	64
	Range	28–82
Tumor stage	I	8
	II	10
	III	28
	IV	16
	Undefined	2
Tumor size (T-stage)	T1 + T2	10
	T3	19
	T4	35
Lymph node metastases (*N*-stage)	N0	16
	N1 + 2	9
	N3	38
	Undefined	1
Distant metastases (M-stage)	M0	46
	M1	16
	Undefined	2
Lauren type	1	34
	2	24
	Undefined	6
Control group		34
Gender	Female	10
	Male	24
Age	Median	51.5
	Range	27–76

**Table 2:** Serum concentrations of proteins in patients with gastric cancer and the control group.

		CXCR2 [ng/mL]	CXCL8 [pg/mL]	CRP [mg/L]	CA19-9 [U/mL]	CEA [ng/mL]
Gastric cancer patients	Minimum	0.000	0.000	0.400	0.000	0.000
	Median	1.449	28.486	16.400	7.875	1.670
	Maximum	4.080	184.054	310.400	1200.000	256.160
Control group (healthy individuals)	Minimum	0.000	0.000	0.200	2.000	0.500
	Median	0.638	6.561	1.050	4.965	1.460
	Maximum	1.895	27.007	5.000	40.970	4.540
*p* (Mann–Whitney test)		*p* < 0.001	*p* < 0.001	*p* < 0.001	0.009	0.797

**Table 3:** Median serum concentrations of assessed proteins in gastric cancer stages.

GC Feature	GC Stage	CXCL8 [pg/mL]	CXCR2 [ng/mL]	CRP [mg/L]	CA19-9 [U/mL]	CEA [ng/mL]
TNM stage	I	33.089	1.260	4.550	5.790	1.740
	II	19.615	0.878	4.500	6.240	1.215
	III	32.709	1.823	17.250	14.815	1.515
	IV	28.486	1.141	52.900	9.605	1.810
*p* (Kruskal–Wallis test)		0.494	0.335	0.044 *	0.087	0.676
T-stage	T1 + 2	29.208	1.260	4.550	5.790	1.380
	T3	23.775	0.940	3.000	5.960	1.240
	T4	32.163	1.802	21.300	13.740	1.860
*p* (Kruskal–Wallis test)		0.124	0.312	0.020 *	0.027 *	0.455
*p* (post hoc Dwass–Steele–Critchlow–Fligner test)	1 + 2 vs. 3			0.809	0.444	
	1 + 2 vs. 4			0.079	0.031 *	
	3 vs. 4			0.061	0.264	
*N*-stage	N0	25.161	1.260	6.550	6.185	1.485
	N1 + 2	23.422	1.802	17.600	11.950	0.940
	N3	30.513	1.449	20.150	13.475	1.915
*p* (Kruskal–Wallis test)		0.754	0.894	0.182	0.034 *	0.076
*p* (post hoc Dwass–Steele–Critchlow–Fligner test)	0 vs. 1 + 2				0.291	
	0 vs. 3				0.032 *	
	1 + 2 vs. 3				0.704	
M-stage	M0	28.620	1.590	10.500	7.495	1.485
	M1	28.486	1.141	52.900	9.605	1.810
*p* (Mann–Whitney test)		0.368	0.450	0.048 *	0.403	0.338

* statistically significant when *p* < 0.05. Abbreviations: GC—gastric cancer, UICC—International Union Against Cancer, T-stage—depth of tumor invasion, *N*-stage—presence of lymph node metastasis, M-stage—presence of distant metastasis.

**Table 4:** Correlations between gastric cancer features and concentrations of biomarkers for gastric cancer patients.

		T	*N*	Staging	CXCR2	CXCL8	CRP	CA19-9	CEA
T	r	1.00	0.55	0.69	0.13	0.18	0.35	0.33	0.15
	*p*		<0.0001 *	<0.0001 *	0.317	0.152	0.005 *	0.007 *	0.229
*N*	r	0.55	1.00	0.73	−0.06	0.05	0.21	0.32	0.21
	*p*	<0.0001 *		<0.0001 *	0.641	0.698	0.091	0.011 *	0.103
Staging	r	0.69	0.73	1.00	−0.01	0.13	0.35	0.27	0.14
	*p*	<0.0001 *	<0.0001 *		0.957	0.319	0.005 *	0.034 *	0.277
CXCR2	r	0.13	−0.06	−0.01	1.00	0.57	0.54	0.16	0.09
	*p*	0.317	0.641	0.957		<0.0001 *	<0.0001 *	0.105	0.400
CXCL8	r	0.18	0.05	0.13	0.57	1.00	0.58	0.21	−0.01
	*p*	0.152	0.698	0.319	<0.0001 *		<0.0001 *	0.042 *	0.957
CRP	r	0.35	0.21	0.35	0.54	0.58	1.00	0.15	0.05
	*p*	0.005 *	0.091	0.005 *	<0.0001 *	<0.0001 *		0.143	0.607
CA19-9	r	0.33	0.32	0.27	0.16	0.21	0.15	1.00	0.21
	*p*	0.007 *	0.011 *	0.034 *	0.105	0.042 *	0.143		0.038 *
CEA	r	0.15	0.21	0.14	0.09	−0.01	0.05	0.21	1.00
	*p*	0.229	0.103	0.277	0.400	0.957	0.607	0.038 *	

* statistically significant when *p* < 0.05. Abbreviations: r—correlation coefficient, *p*—*p* value, GC—gastric cancer. UICC—International Union Against Cancer, T-stage—depth of tumor invasion, *N*-stage—presence of lymph node metastasis.

**Table 5:** Logistic regression test results in relationship between risk factors and gastric cancer (GC) occurrence.

Univariate Logistic Regression Results				
	*p*	OR (Odd Ratio)	95% C.I. (Confidence Intervals)	
CXCR2	0.000	3.923	1.981	7.766
CXCL8	0.000	1.125	1.067	1.186
CRP	0.005	1.457	1.118	1.900
CA19-9	0.121	1.027	0.993	1.061
CEA	0.262	1.118	0.920	1.357
**Multivariate Logistic Regression Results**				
	**Full Logistic Model**			
	*p*	OR (Odd Ratio)	95% C.I. (Confidence Intervals)	
CXCR2	0.864	0.877	0.196	3.932
CXCL8	0.005	1.172	1.048	1.311
CRP	0.027	1.340	1.034	1.737
CA19-9	0.221	1.042	0.976	1.112
CEA	0.412	1.252	0.733	2.138
**Logistic Model after Variable Elimination**				
	*p*	OR (Odd Ratio)	95% C.I. (Confidence Intervals)	
CXCL8	0.002	1.137	1.048	1.234
CRP	0.041	1.279	1.010	1.620

**Table 6:** Diagnostic criteria for biomarkers tested.

	Diagnostic Sensitivity (%)	Diagnostic Specificity (%)	PPV (%)	NPV (%)	ACC (%)
CXCL8	67	94	96	60	77
CXCR2	63	85	89	55	70
CRP	67	100	100	62	79
CA19-9	70	59	76	51	66
CEA	20	100	100	40	48
CXCL8 + CXCR2	81	85	91	71	83
CXCL8 + CRP	83	94	96	74	87
CXCL8 + CA19-9	89	53	78	72	77
CXCL8 + CEA	72	94	96	64	80
CXCR2 + CRP	78	85	91	67	81
CXCR2 + CEA	69	85	90	59	74
CXCR2 + CA19-9	84	91	95	76	87

Abbreviations: PPV—positive predictive value, NPV—negative predictive value, ACC—diagnostic accuracy.

## Data Availability

The data presented in this study are available on request from the corresponding author.
